# Flow cytometry based-FRET: basics, novel developments and future perspectives

**DOI:** 10.1007/s00018-022-04232-2

**Published:** 2022-03-30

**Authors:** JiaWen Lim, Moritz Petersen, Maximilian Bunz, Claudia Simon, Michael Schindler

**Affiliations:** Institute for Medical Virology and Epidemiology of Viral Diseases, University Hospital Tübingen, Tübingen, Germany

**Keywords:** FACS, Förster resonance energy transfer, FLIM, Fluorescence proteins, Molecular interactions, Protein interactions

## Abstract

Förster resonance energy transfer (FRET) is a widespread technology used to analyze and quantify protein interactions in multiple settings. While FRET is traditionally measured by microscopy, flow cytometry based-FRET is becoming popular within the last decade and more commonly used. Flow cytometry based-FRET offers the possibility to assess FRET in a short time-frame in a high number of cells thereby allowing stringent and statistically robust quantification of FRET in multiple samples. Furthermore, established, simple and easy to implement gating strategies facilitate the adaptation of flow cytometry based-FRET measurements to most common flow cytometers. We here summarize the basics of flow cytometry based-FRET, highlight recent novel developments in this field and emphasize on exciting future perspectives.

## Introduction

Förster resonance energy transfer (FRET) is a process in which a donor fluorophore in its excited state transfers its excitation energy non-radiatively to an acceptor fluorophore via dipole–dipole interactions, subsequently leading to the emission of the fluorescence of the acceptor. Since its discovery in 1948 [1, 2], FRET has developed to be a powerful tool to detect interactions between molecules and especially proteins, opening new possibilities in the broad field of biomedical research. Especially since the 1990s FRET-techniques have been implemented and continuously developed as a broad application in life sciences [3]. A comprehensive overview on the physical background of FRET can be found in literature [4–6]. FRET-measurements mostly depend on the use of a microscope, where certain limitations apply. For example, a relatively low throughput rate, special equipment, low signal-to-noise and the absolute necessity for careful normalization and data analysis to exclude false-positive signals. To overcome these problems, since the mid-90 s, a combination of FRET with flow cytometry has been gradually developed [7]. In this review, we intend to introduce FRET briefly with the factors to consider choosing optimal FRET pairs and the different FRET measurement techniques. We then focus on flow cytometry based-FRET, explaining the working principle, the advantages and drawbacks of this approach as well as the respective application in cell-based studies. Importantly, as this review is about FRET in the context of cell biology, we do not discuss on the multiple possibilities FRET offers in the field of recombinantly expressed and purified proteins.

## FRET

**Fig. 1:**
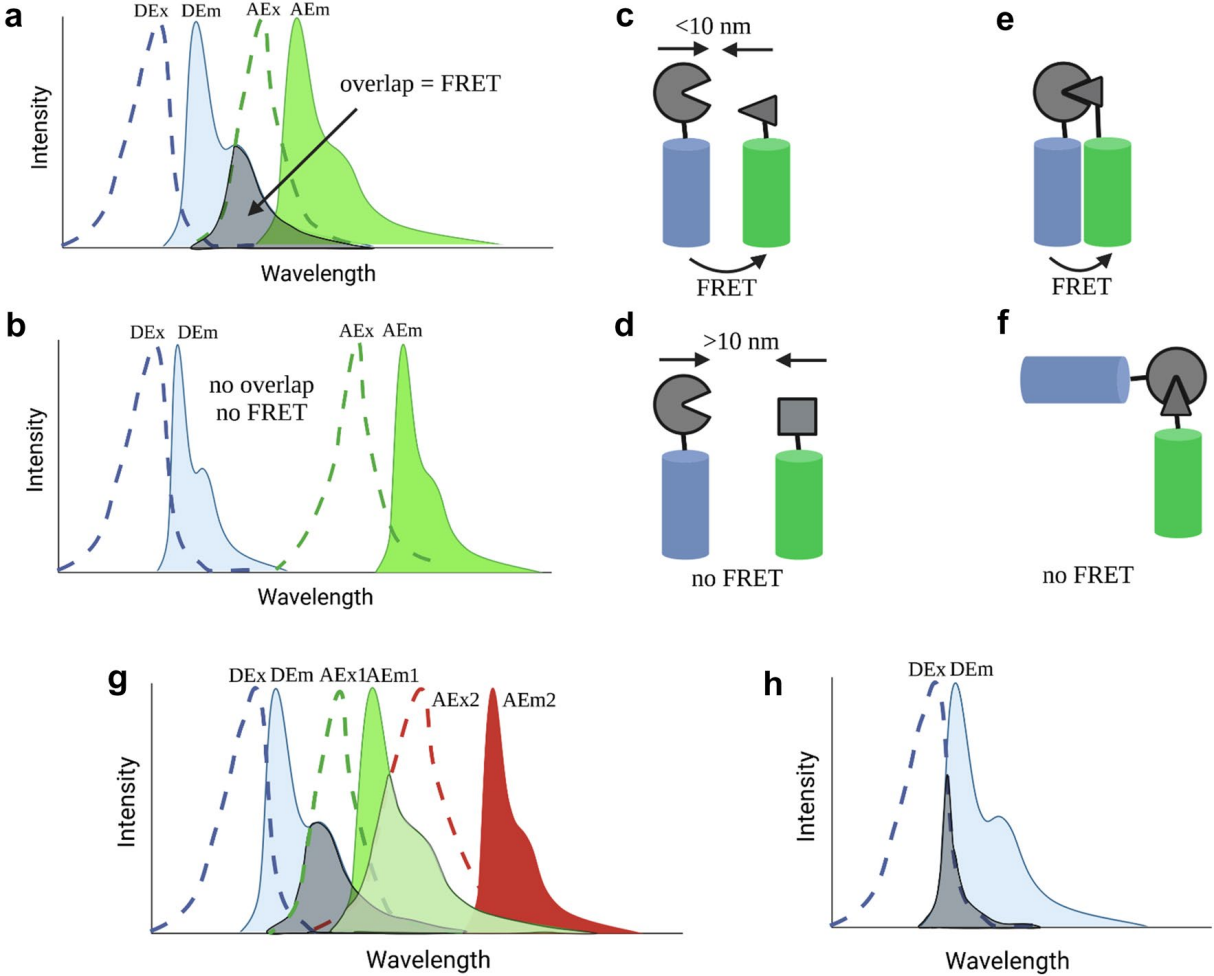
The principle of FRET. Basic conditions needed for FRET to occur include: **a** The overlapping of donor’s emission spectra with acceptor’s excitation spectra (grey area); **b** no FRET if the donor’s emission spectra are separated from acceptor’s excitation spectra; **c** FRET occurs when Förster radius is < 10 nm; **d** No FRET when Förster radius is > 10 nm; **e** FRET occurs at mutual molecular orientation of donor and acceptor; **f** No FRET when orientation of donor and acceptor are different; **g** In three fluorophore FRET, the emission spectra of the donor is overlapping with the excitation spectra of acceptor 1 (grey area), acceptor 1 which is also the donor of acceptor 2, overlap its emission spectra with excitation spectra of acceptor 2 (pale green area); **h** The excitation spectra of donor overlap with emission spectra of donor in single fluorophore FRET (grey area). *DEx* donor excitation, *DEm* donor emission, *AEx* acceptor excitation, *AEm* acceptor emission. Figure created with BioRender.com

In general, for FRET to occur between donor fluorophore and acceptor fluorophore, three basic conditions must be met [8]:

1. Suitable pairing of donor and acceptor: the energy of emitted light of the donor fluorophore must be absorbed by the acceptor fluorophore. Practically, the emission spectrum of the donor has to overlap with the excitation spectrum of the acceptor (Fig. 1a, b).
2. FRET donor and acceptor must be within the Förster radius: FRET only takes place within a distance from 0.5 to 10 nm [5, 9], depending on the fluorescent molecules (Fig. 1c, d).
3. FRET donor and acceptor must have a suitable orientation (Fig. 1e, f): as FRET does not transfer energy via photons but relies on dipole–dipole interaction instead [6, 9].

FRET, however, is not necessarily restricted to the use of two fluorophores. Different groups have shown that three fluorophores can be used, too (Fig. 1g), therefore enabling two FRET measurements at the same time and hence interaction of protein complexes [10, 11]. Another possibility is the use of the same fluorophore with highly overlapping excitation and emission as donor and acceptor (homo FRET, Fig. 1h). In any case, selection of the optimal donor and acceptor FRET pairs is essential for an optimal FRET efficiency.

## FRET efficiency

FRET is characterized by FRET efficiency (*E*). According to Eq. 1, FRET *E* is highly dependent on the distance between donor and acceptor fluorophores that come into close proximity. The closer the distance between donor and acceptor is, the more energy can be transferred resulting in more efficient FRET [12].

1 $$E = \frac{{R_{0}^{6} }}{{R^{6} + R_{0}^{6} }}$$

in which *R* is the actual distance between donor and acceptor dipoles; *R*_0_ indicates the “Förster distance” (or radius), the distance at which the probability for FRET to occur is 50% [4]. The spectral properties of the donor and acceptor is affecting the magnitude of the *R*_0_ which can be evaluated based on the orientation between fluorophores ($k^{2}$), quantum yield of the donor ($\phi_{{\text{D}}}$), refractive index of the medium surrounding the fluorophores (n) and the spectra overlap integral of donor and acceptor (J) as shown in the Eq. 2 below.

2 $$R_{0} \left( {{\text{nm}}} \right) = 0.02108\sqrt[6]{{(k^{2} \phi_{D} n^{ - 4} J)}} \to J = \mathop \int \limits_{0}^{\infty } F_{D} \left( \lambda \right)\varepsilon_{A} \left( \lambda \right)\lambda^{4} d\lambda$$

$F_{D} \left( \lambda \right)$ is the normalized fluorescence emission of the donor (wavelength dependent); $\varepsilon_{A}$ is the extinction coefficient (in ${\text{M}}^{ - 1} \;{\text{cm}}^{ - 1}$) of the acceptor (wavelength dependent); ${\uplambda }$ is the wavelength. $k^{2}$ is the orientation between fluorophores and often assumed to be 2/3 corresponding to a random orientation as the exact $k^{2}$ of FPs is unknown. A different $k^{2}$ value would give a different $R_{0}$ but the trends between the different FRET pairs stay the same. Similar excitation and emission spectra are often shared by fluorescent proteins of the same spectral class. However, they may have different extinction coefficients ($\varepsilon$) and quantum yields ($\phi$). Based on Eq. 2, when the donors have similar emission spectra, the FP with a larger $\phi_{{\text{D}}}$ (quantum yield) is predicted to be a better donor. An acceptor that has a larger extinction coefficient compared to another acceptor with similar excitation spectra is expected to be a superior acceptor due to the larger overlap integral. The *R*_*0*_ of some commonly used fluorophores in FRET have been determined and reviewed previously [13]. Additionally, FPBase (https://www.fpbase.org) is a highly recommended online tool for *R*_*0*_ calculation [14].

Calculating FRET efficiency seems more complicated than using “easier” proxies for FRET, as for instance relative increase in fluorescence intensity or the number of FRET-positive cells. However, using FRET efficiency is an unbiased parameter exactly quantifying FRET, independent of the used instrumentation and most importantly corrected for bleed-through emission.

## Optimal donor and acceptor FRET pairs

Proteins, which contain aromatic amino acids harbor intrinsic fluorescence in the UV-range and FRET can occur between tyrosine and tryptophan. Most proteins contain a variety of these amino acids resulting in a huge background of protein fluorescence, especially in a cellular environment. Fluorescent proteins (FPs) harbor an additional specific fluorophore which leads to fluorescence in the visual range. FPs are commonly used in fluorescence microscopy and flow cytometry. For exogenous expression, cells are transfected with plasmids to express FPs fused to specific proteins of interest (POIs). Most FPs were originally derived from different sea organisms such as corals or jellyfish [4], one of the first and most popular FP is GFP (green fluorescent protein). As of today, a great number of different FPs have been developed, providing a bevy of different options regarding absorbance and emission spectra, pH- or oxygen sensitivity, as well as the quantum yield or the rate of events per absorbed photons [15, 16]. Contemporary fluorescent proteins cover all of the visible spectrum from blue to red (BFP, CFP, GFP. YFP, mRFP and the far-red range) [17], most of them being originally derived from GFP [4]. Red fluorophores in particular have gotten more attention in recent years due to some favorable properties as they show a reduced autofluorescence [18] as well as a larger Stoke’s shift, which defines the difference in wavelength between excitation and emission maxima [4]. Furthermore, red fluorophores are better suited for live cell imaging, as usage of longer wavelengths result in less cellular phototoxicity. With regard to the FRET requirements mentioned above, several factors, like spectral and biochemical properties of the FPs should be considered and are summarized in Table 1.

**Table 1:** Considerations during donor and acceptor FRET pair selection

Factors	Considerations
FRET efficiency	Distance between fluorophores Orientation between fluorophores Laser/filter of the measuring instruments Spectral properties - Spectral overlap - Cross excitation, bleed-through or crosstalk of donor and acceptor fluorophores
Biochemical properties	Oligomeric state of the fluorophores Folding and maturation time of the fluorophores Photostability of fluorophores pH sensitivity of fluorophores
Probes labeling	For exogenous proteins - FP tagging site (N-, C-, or within POI) - With or without linker - Length of linker For endogenous proteins - Fluorophore conjugated antibodies - SNAP/CLIP tags (also for exogenous proteins) - CRISPR/Cas9

To excite and measure FRET, two different laser-filter systems are used, in which the laser functions to excite a fluorophore and the filter to detect its emission.

A one laser system is usually used to measure homo-FRET in which the fluorescence polarization is measured with a polarizer and only one filter is used. To measure fluorescence lifetime (FLIM, will be explained below), only one laser is used, too.

A combination of two lasers and two filters is employed to measure hetero-FRET [19]. It is the most widely used and commonly known as 3-filter FRET for it allows measurements of three different combinations of excitation and emission wavelengths:

(i) A combination of donor-specific excitation and donor-specific emission.
(ii) A combination of acceptor-specific excitation and acceptor-specific emission.
(iii) The FRET channel, a combination of donor-specific excitation and acceptor-specific emission.

The overlap of the emission spectra of donor with acceptor excitation spectra is one of the key elements for FRET to occur. However, in some cases, the cross excitation (stimulation of acceptor with donor exciting light), the bleed-through (donor fluorescence spill over into the emission of acceptor) as well as the crosstalk between acceptor and FRET signal due to their similar emission spectra could be problematic. This can be avoided or reduced by choosing FRET pairs based on the laser and filter set of the available instrument that allow an optimal excitation of the donor without or with compensable cross excitation from the acceptor. Furthermore, the acceptor emission should be detected with filters that have no or minimal donor bleed-through. It is also possible to select FRET pairs from fluorophores in which the spectra are separated to a maximum (large Stoke’s shift). However, it has to be noted that reducing the spectral overlap integral will also result in reduced FRET.

Another thing to consider is the oligomeric nature of fluorescent proteins. Furthermore, oligomerization might also happen due to local high chromophore concentrations, when FPs are present in confined regions like the plasma membrane [20]. To avoid some of these issues, a numerous amount of monomeric fluorescent protein variants has been introduced and reviewed previously [13, 21, 22].

In addition, the folding, maturation time, photostability and pH sensitivity of FPs should also be considered as they could affect the FRET efficiency. FPs with good folding kinetics may enhance the quantum yield [23, 24] and the fluorescence proteins in a FRET pair with similar and fast maturation may further enhance the FRET performance [23, 25–27]. The use of brighter and more photo-stable fluorophores is advantageous for FRET measurements [17, 26]. The use of pH sensitive fluorophores may also reduce the FRET efficiency [28]. Some commonly used FRET pairs had been reviewed previously based on the mentioned factors [12, 13]. As an example, CFP and YFP which have been the most popular fluorophores for long [29] are less utilized nowadays. The reason is that CFP and YFP have some limitations in their applicability [30], such as their dependence on pH and the relatively low quantum yield of CFP, which renders it non-optimal as a FRET donor. A feasible alternative, therefore, could be the use of Clover/mRuby2 as FRET-pair, a green and a red fluorophore, which show increased Förster distance and yield better possibilities for the detection of fast molecular interactions [4, 31].

The strategy of fusing a FP to the amino (N-) or the carboxy (C-) terminus of POIs to study their biological functions in living cells is widely employed in the field. Most of the fluorescent proteins are relatively large molecules of about 240 amino acids with a mass of 25 kDa [17]. However, such tagging may impair the native properties of these POIs, as the fused fluorescent protein may alter the folding, functionality and interaction of a target protein [4]. To control for this, it is advisable to analyze FRET signals with both, C-terminal and N-terminal tags [17, 32]. Besides, the fluorescent proteins could be tagged to the POIs via a linker sequence consisting of up to 30 amino acids [17], to guarantee sufficient motility of the FPs. However, one should be aware that inserting a linker of different sequences may shift the distance and the orientation of the fluorescent protein which might hence affect FRET efficiency. On top, due to the unpredictable complex formation between the two proteins, the fluorophores might be orientated in a turned-away-position, resulting in an interaction without FRET. As the N- and C- termini of FPs are often quite proximate [33], swapping the FP might not result in FRET, even though an interaction is expected. Then, another possibility is to insert the FP into the target protein between functional domains, which could result in FP positioning alongside the target protein, without disrupting it [34–36]. In this case, structural information of the POI is beneficial to assure insertion does not disrupt the POI’s structure [34].

Fluorophore conjugated antibodies are a popular tool to study interactions with endogenous cellular proteins [4], as they show high affinity and specificity towards their binding domain. However, due to their considerable size of ~ 150 kDA and bivalent binding property, they also entail certain problems as they tend to form artificial clusters, thus tampering with FRET measurements. Furthermore, the use of antibodies largely precludes the possibility to analyze living cells, as for internal protein staining cells need to be fixed and permeabilized. Today, such an alternative is the use of SNAP/CLIP tags, using derivatives of human DNA repair proteins which are then co-expressed with the protein of interest as SNAP/CLIP-tag, leaving the possibility for side-specific tags [4, 37]. After that, a cell-permeable fluorescent molecule, linked to *O*^6^-alkylguanine (in case of SNAP) or *O*^6^-benzylguanine (in case of CLIP) can be attached to this complex [17]. When used together, SNAP and CLIP can thus function to label two different proteins with fluorophores. Additionally, this approach offers the possibility to introduce many different fluorophores to one target molecule [29].

Another possibility that arose with the CRISPR/Cas9 technology is insertion of FPs into endogenous proteins. This would allow to perform FRET based on FP-tagging without the necessity, limitations and problems of transfecting cells to overexpress exogenous proteins.

## Possible approaches to measure FRET

**Fig. 2:**
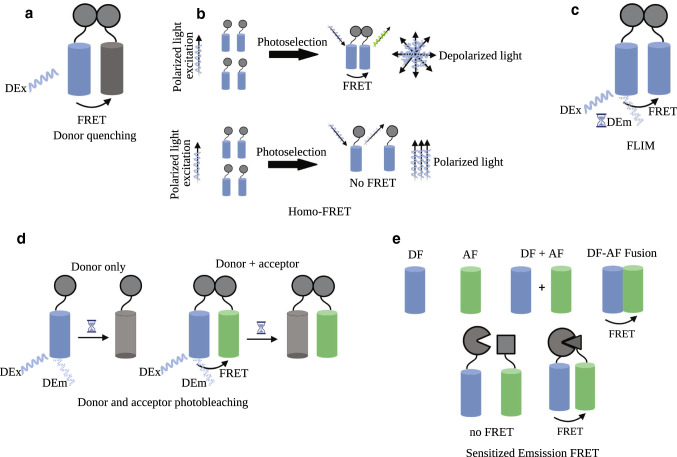
Different approaches to measurement FRET. There are different approaches to measure single fluorophore homo-FRET (top panel) and hetero-FRET (bottom row). **a** Donor quenching: the non-fluorescent acceptor releases the acquired energy non-radiatively. **b** Homo-FRET: fluorescence molecules excited by polarized light. Energy transfer between two identical fluorophores, polarized light becomes depolarized due to the different orientation indicating homo-FRET. The light remains polarized in the absence of energy transfer. **c** FLIM: the energy transfer is monitored via the fluorescence lifetime of the donor fluorophore. **d** Donor and acceptor photobleaching: the photobleaching of donor is monitored in presence or in absence of acceptor. **e** Sensitized emission FRET: The control of the measurement includes donor fluorophore (DF) only (negative control, no FRET), acceptor fluorophore (AF) only (negative control, no FRET), donor fluorophore + acceptor fluorophore (negative control, no FRET), donor–acceptor fluorophore fusion (positive control, FRET). FRET only occurs when the two FP tagged target proteins come into close proximity. Figure created with BioRender.com

Over 20 different imaging techniques to determine FRET have been suggested [2, 5, 38, 39], most of which rely on the use of fluorescence microscopy. Several possible FRET approaches that involve single fluorophore (homo-FRET) or multiple fluorophore (hetero-FRET) will be outlined in brief and summarized in Fig. 2.

## Single fluorophore FRET

In case of FRET, the fluorescence emission from the donor is quenched. This donor quenching can be monitored as the non-fluorescent acceptor is releasing the energy transferred from the donor non-radiatively [12]. This approach allows FRET measurement with multiple FRET pairs at the same time, as the spectra areas occupied by the acceptors’ emission become available. Donor quenching can also be measured in hetero-FRET.

Polarization anisotropy imaging uses polarized light, exciting only the fluorescence molecules aligned parallel to the polarization vector (photoselection). If the molecules do not rotate, fluorescence emission maintains this parallel orientation, the fluorescence is anisotropic [5]. Thus, anisotropy of a molecule is an indicator of its orientation. Anisotropy imaging then compares the orientation of the excited and of the emitting molecules. Without FRET, there is virtually no difference between both. However, upon FRET, the excited and the emitting molecule are no longer the same and a substantial decrease in the correlation of their orientation can be observed [34]. This is then used to determine the rate of FRET. Of note, polarization anisotropy is the only technique capable of detecting FRET between two identical fluorophores, and is therefore called homo-FRET [12]. As the spectra of donor and acceptor are exactly the same here, none of the aforementioned techniques allow to measure homo-FRET. However, homo-FRET based on polarization anisotropy is not applied broadly, most likely due to the complicated assay read out and expert knowledge necessary to perform reliable measurements [40].

Fluorescence lifetime imaging microscopy (FLIM) allows FRET determination by investigating how long a fluorophore remains in an excited state, suggesting a certain FRET efficiency [5]. It was first implemented by Wang et al. in 1989 [41]. Characteristically, each fluorochrome’s emission is reduced after being excited, thus allowing the determination of a fluorescence lifetime. As FRET alters the state of excitement of both fluorophores, reducing the donor’s fluorescence lifetime and increasing the acceptor’s, FLIM allows to determine FRET efficiency. It has proven to be a very potent tool, for it lacks certain limitations of other FRET techniques, such as sensitivity to signal cross-contamination or photobleaching. Thus, it is the most accurate method to measure FRET in living cells [5].

## Multiple fluorophores FRET (Hetero-FRET)

Donor and acceptor photobleaching rely on the fact that an excited fluorophore is more likely to undergo a process of covalent modification, rendering it incapable of undergoing the excitement-emission process again. Molecules involved are often reactive oxygen species (ROS) which are known for their ability to irreversibly change biomolecules. Here, the effect that the presence of an acceptor reduces donor photobleaching, is used to calculate FRET efficiency by comparing donor photobleaching in presence and in absence of the acceptor fluorophore. However, this method is somewhat prone to limitations, as it requires relatively long excitation time to achieve photobleaching, potentially challenging the cell’s homeostasis and affecting cellular viability [5].

In a different technique, only the sensitized emission of the acceptor molecule is measured. This is the simplest way to measure FRET and it is the main technique also exploited in the context of flow cytometry based-FRET. Therefore, different samples have to be prepared to serve as a control for the intended measurement [32, 42]:

(i) Donor fluorophore only.
(ii) Acceptor fluorophore only.
(iii) Donor and acceptor fluorophore fused together.
(iv) Donor and acceptor fluorophore separately.

Although this method is relatively easy, its main disadvantage when done via fluorescence microscopy is that there is a huge fluorescence crosstalk between the various imaging channels, leading to a high background and a low signal-to-noise ratio. To correct for this, extended image processing and normalization is necessary [5].

## Combination with flow cytometry

FRET-measurements mostly depend on the use of a microscope, where certain limitations apply. For instance, a relatively low throughput rate, special equipment, low signal-to-noise ratio and the absolute necessity for careful normalization to exclude false-positive signals. To overcome these problems, since the mid-90s, a combination of FRET with flow cytometry has been gradually developed, called FACS-FRET (FACS is Fluorescence activated cell sorting) [7].

The major FRET technique used in FACS measurements is sensitized emission [19], even though donor-quenching was relatively early established as a robust technique to assess FRET by FACS [43]. Another approach is FLIM based FACS-FRET, which is restricted to special flow cytometers. As there has already been an excellent review on this issue [44], this manuscript will focus on the intensity-based approach, instead.

## Possible applications of FACS-FRET

After its first description, even though FACS-FRET soon showed to have major advantages compared to other techniques [7, 45], it took some 15 years for it to find broader application, yielding more than 300 results at PubMed as of today. FACS-FRET proved tremendously successful when it comes to the detection of molecular interaction in cells. Essentially, it is able to measure FRET in virtually all cellular compartments [32]. So far it has been used not only to study protein interactions in the cytoplasm, as for example in the detection of cellular pathways [46] or protein self-assembly [47] but also at the plasma membrane or different membranes like the endoplasmic reticulum  [32] and in the analysis of receptor oligomerization [34, 48]. When FRET between fluorophores in the same membrane subdomain is measured, the work of Winkler et al. [49] indicates that random FRET without direct molecular interaction occurs more often as there is much less space for the molecules to diffuse freely. To determine FRET-positive cells, this has to be taken into consideration, although the effect was found to be relatively weak.

Additionally, Suffner et al. demonstrated that FACS-FRET can also be used to study the oligomerization of viral proteins within cells, in this case the S protein of the hepatitis B virus, and its role in the formation of subviral units [50]. The technique has also been used to study interactions between proteins of both virus and host [32, 51].

FACS-FRET, however, is not only restricted to mammalian cells but can also be used to study other organisms. Voyton et al., for example, have shown that the technique is very well applicable to protozoa like *Trypanosoma brucei* [52]. An earlier paper describes a FACS-FRET based experiment approach to study *Escherichia coli* [53].

Another approach FACS-FRET was used for, is to study the influence of individual amino acid positions on protein interaction and function. Papers pursuing this approach have been presented, for example, by Hagen et al. [42] or Winkler et al. [49]. In addition, the technique allows to study the influence of pharmacological treatment on molecular interactions as demonstrated by Trümper et al. [30] or van de Wiel et al. [54]. Lastly, Doucette et al. have shown that FACS-FRET is suited to measure not only inter- but also intramolecular interactions [55].

**Fig. 3:**
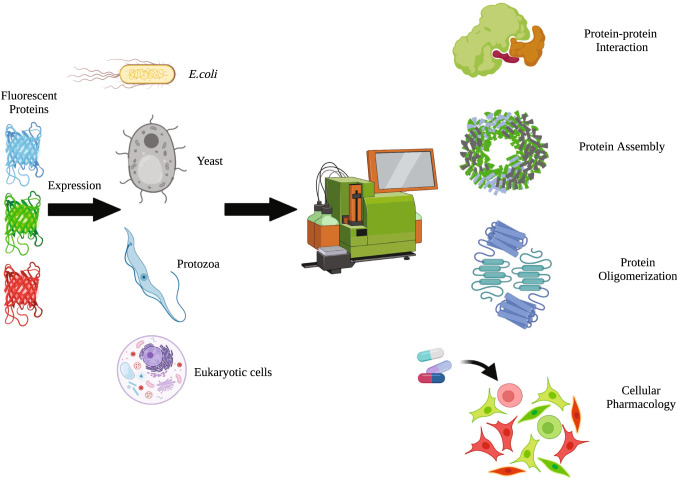
Application of FACS-FRET in cell-based studies. Fluorescently labeled proteins of interest could be expressed in different expression systems including *E. coli*, protozoa, yeast and eukaryotic cells and subjected for FACS-FRET analysis for the study of protein–protein interactions [32, 42, 46, 49, 51, 53, 59, 60], protein assembly [47], protein oligomerization [34, 48, 50] and cellular pharmacology [30, 54]. Figure created with BioRender.com

As explained in the introduction, FRET as a biophysical phenomenon has been known for long and became popular in life sciences when it was possible to link FRET to a molecular process of interest, initially mainly protein interaction. However, in the meantime, many additional applications of FRET have been realized, for instance activity of enzymes, apoptosis as proxy of caspase cleavage, proximity of probes to membranes, or intracellular signaling, i.e. cGMP or calcium signaling, to name just a few examples. These measurements are usually realized by specific FRET-reporters and read-out is microscopy [56–58]. In the future, such reporter assays might also be adapted to the flow cytometry based-FRET measurements, with the advantages discussed (Fig. 3).

## Constraints

Despite the huge potential of FACS-FRET, making it a powerful tool in contemporary biomedical research, it is not without limitations. Most of these arise from the natural constraints of FRET itself, such as the fact that cells are transfected to overexpress the FP-tagged target proteins, which could result in artefacts like altered subcellular localization and function, activation of the ERAD (endoplasmic reticulum associated degradation) and cytotoxicity [17]. Therefore, compared to antibody-based FRET, a major constraint is that an exogenous expression of target proteins is required [3], an obstacle that could be overcome by FP-tagging of endogenous host cell proteins via CRISPR/Cas9.

In addition, absence of FRET does not necessarily indicate a lack of interaction [61]. This is due to the inherent property of FRET, that for an efficient energy transfer a very small distance between both fluorophores is necessary. This distance can be much higher than 10 nm for instance due to the use of chromophore labeled antibodies and their inherent size, or distance increasing parameters as for instance cellular organelles and membranes resulting in a suboptimal FRET signal [30]. On the other hand, even positive FRET signals have to be evaluated carefully, as fluorophores show a certain tendency to self-associate, which may consequently lead to artificial increases in FRET [4].

Another disadvantage of FACS-FRET is that it only allows fluorescence measurement on a cell-by-cell basis. This, of course, is due to the functioning of a flow cytometer, only allowing single-cell measurements. Along with this problem comes that, although living cells can be measured, detachment of adherent cells from their substrate is a prerequisite to the measurement, therefore ruling out measurements of solid tissue [2]. Moreover, the necessary trypsinization of the cells could also impair FRET [62].

FACS-FRET does not detect heterogeneity within cells, as the entire spatial information gets lost [19]. To detect sub-cellular distribution of fluorophores, therefore, fluorescence microscopy is still the gold standard today and even used in FACS-FRET experiments [32]. However, as already mentioned, imaging flow cytometry could set new standards here.

Finally, a major constraint of flow cytometry based-FRET, for long, was its complexity and the lack of a standardized approach. However, in recent years there has been major progress and development, thus increasing popularity of FACS-FRET. Here, an important milestone was the design of an easy to adapt FACS-FRET assay by Banning et al. [32], allowing for a simple reproduction in different experimental settings. Moreover, it facilitated quantification and statistical analysis.

To further simplify the evaluation of FRET data, another crucial improvement has been the development of automated software-based FRET-calculation. Von Kolontaj et al. presented a novel algorithm in 2016, allowing for an automatic detection of T cell activation as measured by FRET post antibody-labeling of the cell surface, thus proposing the first automated flow cytometry based-FRET assay method suited for high-throughput screening [61].

## Prospects in the use of FACS-FRET

Despite all this, considering the virtues of FACS-FRET, the technique has potential for further developments and broad usage in the future.

First of all, FACS-FRET has the major advantage that given the nature of a cytometer, it is a fast, high throughput technique. This has been shown very impressively by van de Wiel et al. [54]. There, they describe how they used the technique to scan 1280 FDA-approved drugs, to find compounds that activate the Farnesoid X Receptor. On top, flow cytometry is not only high throughput in term of cell numbers analyzed, but also advantageous when measuring kinetics [63], for instance assessing FRET over time after a certain stimuli, With regard to its quality, a recent study found virtually no relevant difference in FRET values between microscopy and FACS-FRET while obviously flow cytometry worked much faster and yielded better results due to the analysis of high cell numbers [55, 64]. Additionally, FACS-FRET has the advantage that the data obtained is much easier to interpret as the technique does not require complex data analysis, background and crosstalk correction and many more as compared to fluorescence microscopy [32]. Therefore, FACS-FRET is very valuable for statistics, too, allowing easy categorization of cells. Banning et al. [32] have suggested a feasible approach to quantify FRET-efficiency as the percentage of FRET-positive cells within a sample, which again correlates with the proximity between the molecules. Positive and negative controls were used to establish a gate allowing easy classification of FRET cells. The percentage of FRET-positive samples, then, is a suggestion for the binding strength between both target proteins. Consequently, this approach has been used by various other groups in the following years, such as Hassinen et al. who successfully showed the homomeric and heteromeric complexes formation within Golgi *N-* and *O-*glycosylation pathways in living cells [59] and Furman et al. established a proteopathic seeding assay to study protein aggregation disorders that arise in neurodegenerative diseases [60].

However, this type of analysis is at best semi-quantitative as it only distinguishes between FRET-positive and negative signals, i.e. the FRET ratio. The FRET ratio is calculated simply by dividing donor-specific excitation and acceptor-specific emission through the donor-specific excitation and donor-specific emission. This calculation is largely instrument dependent, hence data generated by different flow cytometers is not comparable. Furthermore, sensitivity is rather low. While this is sufficient when the question is to assess if there is a robust interaction or not, it does not provide the possibility to calculate FRET parameters and to quantify FRET. To achieve that, different approach have been presented [19, 63]. Hochreiter and colleagues [19] use a three-filter based FACS-FRET to establish a saturation curve of apparent FRET efficiencies (DFRET) and describe how they combined it with a mathematical algorithm, allowing a quantitative determination of FRET_max_, the donor acceptor ratio z as well as the interaction strength *K*_a_^app^, which correlates with the affinity constant *K*_a_. The approach is insofar remarkable as it allows the determination of all three of these parameters at the same time, whereas previously, the values had to be obtained separately. FRET_max_ is a measure of particular interest here, as it relates to the actual distance between donor and acceptor. Later, they confirmed that the predicted value for *K*_a_^app^ was indeed similar to the values obtained by in vitro measurement [65].

Again, the combination with flow cytometry proved crucial for their approach as it allowed the measurement of a sufficient number of cells to establish the saturation curve, which would not have been feasible with different methods like microscopy. A similar approach using another mathematical model had also been described previously [66].

As already mentioned, another novel attempt to further expand the possible applications of FACS-FRET is the use of CRISPR/Cas9, potentially opening the doors to studying not only exogenous but also endogenous proteins by FP-tagging. Since the examination of endogenous proteins is far more relevant, the use of CRISPR/Cas9 could well become a very important improvement in the use of FACS-FRET, increasing the technique’s possibilities even further.

Other exciting developments opening new avenues to FACS-FRET are technological advances in flow cytometry. Imaging flow cytometry allows to determine not only if FRET occurs due to the measured FRET signal but also to localize the interaction to a specific subcellular compartment, overcoming one of the major disadvantages of FACS-FRET as compared to microscopy [67]. Recent implementations of high-throughput flow cytometry might not only render this technique high-throughput in terms of individual cells measured per sample, but also related to the total number of samples that can be processed in a reasonable time-frame [68]. By this, it can be envisioned that FACS-FRET finds its way to rational-based screening of protein interaction inhibitors. Finally, one recent exciting study exemplifies the adaptation of FACS-FRET to spectral flow cytometry, which allows much more precise single-cell determination of FRET and hence robust sorting of FRET-positive cells [69].

## Conclusion

To conclude, it should be noted that FACS-FRET is a powerful tool not only in the detection of protein–protein interactions in living cells in different compartments but also with regard to their functionality, pharmacological treatment as well as conditions of stress. The technique is, moreover, not merely restricted to mammalian cells, as different organisms like viruses or protozoa can be studied, too. The current trends outlined above show the potential of FACS-FRET as a research tool in cell biology and the many related disciplines. It will be exciting to further develop and apply this technique in the future.

## Data Availability

Not applicable.
